# Properties of Sound Absorption Composite Materials Developed Using Flax Fiber, Sphagnum Moss, Vermiculite, and Sapropel

**DOI:** 10.3390/ma16031060

**Published:** 2023-01-25

**Authors:** Daira Sleinus, Maris Sinka, Aleksandrs Korjakins, Vaira Obuka, Vizma Nikolajeva, Raitis Brencis, Estere Savicka

**Affiliations:** 1 Institute of Polymer Materials, Faculty of Materials Science and Applied Chemistry, Riga Technical University, 3/7 Paula Valdena Street, LV-1048 Riga, Latvia; 2 3D Concrete Printing Laboratory, Institute of Materials and Structures, Riga Technical University, 1 Paula Valdena Street, LV-1048 Riga, Latvia; 3 Department of Building Materials and Products, Faculty of Civil Engineering, Riga Technical University, 6A Kipsalas Street, LV-1048 Riga, Latvia; 4 Department of Environmental Science, University of Latvia, 1 Jelgavas Street, LV-1004 Riga, Latvia; 5 Department of Microbiology and Biotechnology, University of Latvia, 1 Jelgavas Street, LV-1004 Riga, Latvia; 6 Scientific Laboratory of Building Materials, Department of Architecture and Building, Latvia University of Life Science and Technologies, 19 Akademijas Street, LV-3001 Jelgava, Latvia; 7 Zaiga Gaile Office, 13/IV Marijas Street, LV-1050 Riga, Latvia

**Keywords:** sapropel, hemp, hempcrete, hemp concrete, environmentally friendly composite materials, sound absorption materials, microbiological stability

## Abstract

To address the need to reduce consumption and pollution in the industrial sector, composite materials were created using a new type of raw materials—organic lake sediments (sapropel) as a binder; sphagnum moss, flax fiber, and vermiculite as a filler. The main application of these composite materials is for sound absorption and moisture buffering, but since they contain bio-based binders and fillers, they also work as carbon storage. Within the framework of this work, a total of 100 samples of composite materials were created. Fungicides—a biocide quaternary ammonium compound and its natural substitute montmorillonite mineral material were also added to the materials to improve microbiological stability. The mechanical sound absorption and microbiological properties of materials were investigated and compared to similar environmentally friendly materials, such as hemp-lime concrete (FHL), hemp magnesium oxychloride composite (MOC), and hemp magnesium phosphate cement (MPC). The results showed that sound absorption and mechanical and microbial properties of the created composite materials are sufficient for their intended use, with flax fiber and vermiculite composites showing more stable mechanical, sound absorbing, and microbiological stability properties than materials containing flax fiber and moss.

## 1. Introduction

As environmental protection issues have become more globally relevant, the need to reduce both industrial consumption and air pollution caused by industry is becoming more and more important. Building construction is one of the sectors where new, more environmentally friendly methods should be implemented, for example, improving the energy efficiency of buildings [1]. It is important not only to make buildings energy efficient, thereby saving resources, but also to use more natural materials in their construction to reduce the ecological footprint of buildings and make buildings more environmentally friendly. A growing tendency in production, including the production of building materials, is the reduction of the use of artificial fibers by replacing them with natural ones [2]. 

In this work, flax fiber, vermiculite, and sphagnum moss were used as a filler in the creation of composite materials. In Latvia, climatic conditions and soils are suitable for the cultivation of flax fiber, and it also has long traditions [3]. On the other hand, vermiculite is a material made from rocks of the silicate group—a certain mica-type mineral. Vermiculite is characterized by a low coefficient of thermal conductivity. In addition, it does not change its properties in the temperature range from −200 to +1100 ℃, which makes it a good thermal insulation material in construction [4]. Moss has also historically been used as an insulation material in log constructions. The logs themselves protect the interior from the hot summer sun and winter frost but laying moss between the logs for thermal insulation is a proven method in craft practice [5]. 

Sapropel was used as a binder in composite materials, which is a partially renewable geological resource [6]. It accumulates through the deposition and decomposition of the remains of dead plants and organisms together with mineral particles. In Latvia, sapropel deposits have been studied in 370 Latvian lakes; they have also been found in many bogs under peat [7]. In general, sapropel has wide application possibilities in agriculture, medicine, mining, chemical industry, heat energy, and construction industry. So far, it is known that in construction, it is possible to use sapropel as an additive for making ceramics, porous material for drainpipes, sapropel concrete binder, concrete filler, adhesive thermal insulation, and chipboards [8]. 

Sapropel’s characteristic hydrophilizing and adhesive ability characterizes its binder properties. It also has good viscosity, plasticity, and absorption [6]. Research on the possibilities of using sapropel as a binder has been conducted not only in Latvia but also in Lithuania and Belarus. Research shows that sapropel as a binder, together with various by-products in the creation of new products, results in materials that are included in the category of thermal insulation materials [9]. In Latvian lakes, in approximately 80% of research cases, the content of organic matter in sapropel dry matter is greater than 60%, which indicates that Latvia has high-value biogenic type sapropel resources with wide possibilities of use [10].

Moisture in building materials can lead to the spread of microorganisms in the materials. During the life cycle of buildings, environmentally friendly materials can be affected by biodegradation caused by the growth processes of microorganisms [11], i.e., rotting and rotting of materials and mold. In the case of moisture damage, even after restoration, some material damage remains in the building structures [12]. Therefore, preventing the transport of potential pollutants is of great importance in preserving the design and construction of buildings. It is important to know what factors contribute to the spread of pollutants, as well as possible ways to prevent the spread [11]. Fungi play the biggest role in the biodeterioration of materials made from natural fibers and in the aerobic degradation of cellulose, which is the main component of natural fibers [13]. The effects of fungi growth on natural components used in composite materials have been analyzed in this study. 

Mathematical models and risk assessments exist to determine the biodegradation risk of materials, but mold species and materials vary. Therefore, the most accurate way to determine the risk of biodegradation is to conduct experiments by growing the most common mold species on samples and studying their development and changes in the materials [14]. In recent years, the use of organic materials in the creation of more environmentally friendly composite materials has been increasing rapidly, and new research is being carried out to improve these materials. The effect of fungi on the sustainability of materials, which is very important in ensuring the sustainability of materials, is not fully understood. Therefore, more practical studies in laboratories are needed [15]. Generally, the growth of the number and diameter of fungal colonies or the released heat, which indicates the metabolic activity of fungi, are used as parameters that determine the size of fungal biomass [16]. In different environments, the distribution of mold varies, even if the humidity and temperature conditions are the same. In general, molds and other microorganisms need moisture, nutrients, and the right temperature to grow [17]. To protect materials from fungi growth, antimicrobial components are used–typically biocide products [18]. On the other hand, negative side-effects of the biocides are being discovered, so it is important that each biocide has specific rules of use based on the activity in different environments or is prohibited altogether, as well as to introduce environmentally and organism-friendly alternatives. Typically used biocide in building materials is quaternary ammonium compounds, which toxicity is very high, especially to aquatic organisms [19]. In this study, we have analyzed the antimicrobial effects of a typical quaternary ammonium compound group biocide and its natural alternative montmorillonite mineral material. Clay minerals have the property to exchange ions, and when modified, their surface changes from hydrophilic to hydrophobic [20].

The aim of this paper was to study the properties of innovative, environmentally friendly sound absorption composite materials, using natural raw materials–organic lake sediments (sapropel) as a binder and sphagnum moss, flax fiber, and vermiculite as a filler. 

## 2. Materials and Methods

### 2.1. Binders, Fillers and Additives Used in the Manufacture of Composite Materials

Untreated organic lake sediments (sapropel) from Lake Pilvelis (Rezekne municipality, Latgale) and Lake Piksteres (Jekabpils municipality, Selija) were used as binders for the development of composite materials under laboratory conditions (Table 1). 

Sapropel samples had an almost neutral environmental reaction (pH). The blue-green algal sapropel of Lake Pilvelis was dark brown with a greenish tinge and a clotted structure. Its moisture content was 94.99% and organic matter content was 84.51% [8]. Lake Piksteres sapropel is brownish black. It has a moisture content of 97.00 and 83.00% organic matter (from the dry matter) and a viscous structure [9].

As fillers, to create composite materials, Sphagnum moss, flax fiber, and vermiculite were used. Sphagnum moss was harvested in 2013 in the vicinity of Cena bog, with flax fiber from “Bisan” manufacturer but vermiculite from “Hortis” manufacturer. 

In addition, a bactericide (biocide) from the Latvian manufacturer “Elvi” was added, the active substance of which is quaternary ammonium compounds. Innovative biocide substitute ALINA Ltd. product “ALINA LIFE^TM^” organoclay coating was also added to the materials. The composition and designations of the created materials are described in Table 2.

The obtained results of sound absorption and microbiological durability tests were compared with 5 previously studied [21] environmentally friendly composite materials. The composition and designations of which are described in Table 3.

### 2.2. Methods for Characterization of Sapropel Samples

The environmental reaction (pH) of the sapropel samples was determined in the laboratory. The moisture and organic matter content in samples was described based on previous studies [6,8].

Determination of the environmental reaction (pH) of sapropel samples: a 1 g sample of sapropel, which was used to determine the pH, was weighed into an 80 mL jar with a lid and dissolved in 50 mL of distilled water, then the jars with the samples were placed on a shaker. After 2 h on a shaker, each sample was filtered through filter paper. The pH of the resulting solution was determined with a pH meter.

### 2.3. Methods of Manufacturing Composite Materials

The composite materials were created manually by mixing the filler and binder in an even mass ratio of 1: 6. Two types of sapropel samples were used as binders in the materials, which were mixed by blending to a homogeneous mass in a ratio of 1: 1. Sapropel was not heat treated prior to incorporation into the materials.

The biocide and ALINA LIFE ^TM^ were added to the materials by two methods, either as an additive to the mass (2% of the total mass of ALINA LIFE ^TM^ or 1% of the total mass of the biocide) or as a coating. The material coating was formed using the same sapropel samples used in the materials in a 1: 1 ratio. Sapropel was mixed into a homogeneous mass. In total, 2% of the total mass of ALINA LIFE ^TM^ or 1% of the total mass of the biocide was added to the mass of sapropel. Montmorillonite mineral material ALINA LIFE is a Cradle to Cradle ™ certified additive that replaces biocidal products. Its technology is based on naturally occurring montmorillonite minerals (phyllosilicates) that have been surface treated to convert the hydrophilic surface to a hydrophobic surface without altering the structure of the montmorillonite mineral. Due to their physicochemical properties, clay has a wide range of applications, from paints and coatings, cosmetics, and personal care to lubricants and detergents. 

Samples for sound absorption and microbiological durability tests were formed by placing the mass in a 30 × 30 × 10 cm gypsum mold designed according to the dimensions of the sound absorption test tube, with a radius of 40 mm for each section of the mold. To obtain the corresponding material thicknesses, two types of pins were used, with which the materials were compacted in the mold sections. Wood plywood was screwed to the bottom and top of the mold to secure the specimens and pins. Once the samples were prepared in the mold, they were placed in an oven at 40 to 105 °C for 12 to 24 h.

Samples for mechanical strength tests were formed by placing the mass in a 16 × 4 × 4 cm mold, leveling, and compacting the mass manually. The prepared samples were kept in the oven at 40 to 105 °C for 12 to 24 h.

### 2.4. Mechanical Strength Testing of the Material

A Zwick Z100 universal testing machine was used for mechanical strength tests, applying pressure at a rate of 10 mm/min and recording the force-deformation diagram during the process. Compressive strength was determined at 10% relative deformations (according to LVS EN 826), bending–at the point of collapse.

### 2.5. Sound Absorption Tests

Sound absorption was determined in an acoustic tube with a diameter of 40 mm, in the frequency range from 250 ÷ 4000 Hz.

The weighted sound absorption coefficient was determined according to the standard LVS EN ISO 11654: 2000 “Acoustics–Sound absorbers in buildings–Sound absorption parameters”. The results were analyzed according to the sound absorption classes specified in the standard LVS EN ISO 11654: 2000. 

### 2.6. Microbiological Stability Tests

**Figure 1:**
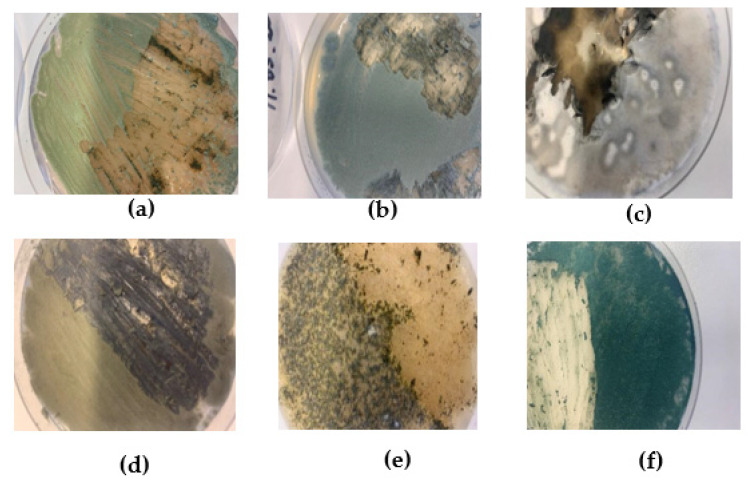
MSCL No., Latin name, and image of the fungi used in the experiment: (**a**) MSCL 1346 *Aspergillus versicolor*; (**b**) MSCL 281 *Penicillium chrysogenum*; (**c**) MSCL 280 *Alternaria alternata*; (**d**) MSCL 258 *Cladosporium herbarum*; (**e**) MSCL 851 *Chaetomium* sp.; (**f**) MSCL 309 *Trichoderma asperellum*.

Microbiological stability tests were performed by artificially inoculating material samples with six fungi from the Microbial Strain Collection of Latvia (MSCL). MSCL number, Latin name, and digital photo without magnification are shown in Figure 1. 

Molds were grown in Petri dishes in malt agar medium, and a suspension of mycelium and spores in sterile (autoclaved at 121 °C for 15 min) distilled water was prepared from each fungus. All six fungi were mixed in equal amounts in one suspension. Each test sample, 40 mm in diameter and 22 mm in average thickness, was moistened with 200 μL of fungal suspension and placed in a plant tissue culture container, followed by an 18-day incubation period. In addition to the inoculated samples, control samples were also placed in the container without the inoculation of the mold. 

Incubation was performed under 99% RH at 20 to 22 °C. In total, 2 mL of sterile distilled water was added as needed to ensure RH conditions in the climate chambers. For samples, every 2 days, at approximately the same time from 12:00 to 13:00 in the afternoon, a visual assessment was performed using an expert rating scale for fungal colony growth [22] (Table 4). The visual evaluation was performed with the naked eye or using a magnifying glass.

After 18 days of incubation, the materials were evaluated microscopically to determine which of the six mold species had developed, as well as to determine which were the dominant colonies.

At the end of the incubation period and after microscopic evaluation, the samples were subjected to pH determination as described in Section 2.2.

## 3. Results and Discussion

### 3.1. Characteristics of the Formed Composite Material Samples

Samples of two types of composite materials were developed under laboratory conditions. Half of the composite samples were formed using flax fiber and vermiculite as filler, and half of the composite samples were formed using sphagnum moss and flax fiber.

Samples were also created for each type of composite material with the addition of an additional biocide or ALINA LIFE. The additional composition was added by two methods—addition to the mass or coating by mixing with sapropel.

**Figure 2:**
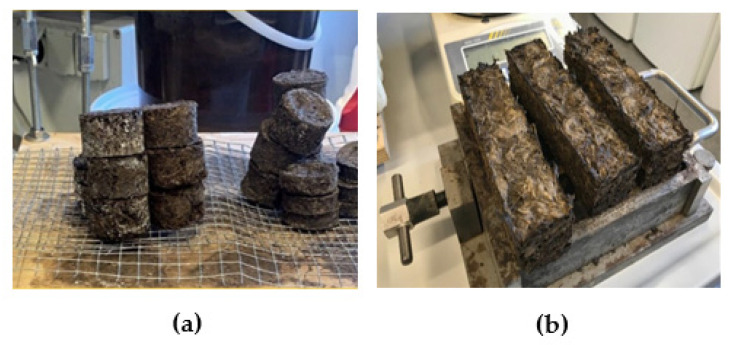
Developed material samples: (**a**) Samples for sound absorption and microbiological resistance tests; (**b**) Samples for mechanical strength tests.

Material samples for sound absorption and microbiological durability tests were 40 mm in diameter in two thickness categories—25 mm in thickness and 15 mm in thickness (Figure 2), and material samples for mechanical tests in 40 mm in thickness, 40 mm in width, and 160 mm in length. 

The pH of the samples for microbiological tests was determined as well as the density of the samples for mechanical tests (Table 5). Both types of composite materials have a low density. The weight of the samples formed for the mechanical tests ranged from 45 to 50 g for sapropel, flax fiber, and vermiculite materials and 35 to 45 g for sapropel, flax fiber, and moss materials. Sapropel, flax fiber, and vermiculite materials were more compacted than sapropel, moss, and flax fiber materials. Materials made with moss were more porous and flexible, so they were not so easy to break.

In composites of sapropel, flax fibers, and vermiculite, the latter forms a small reflective pattern, which, on a large scale, would give the material not only a functional but also a decorative character. On the other hand, sapropel, flax fiber, and sphagnum moss materials would have a larger natural moss pattern, which also gives a decorative element. The fungicides did not cause any visual change in the impurity, but the sapropel coating obscured the texture of the samples.

**Figure 3:**
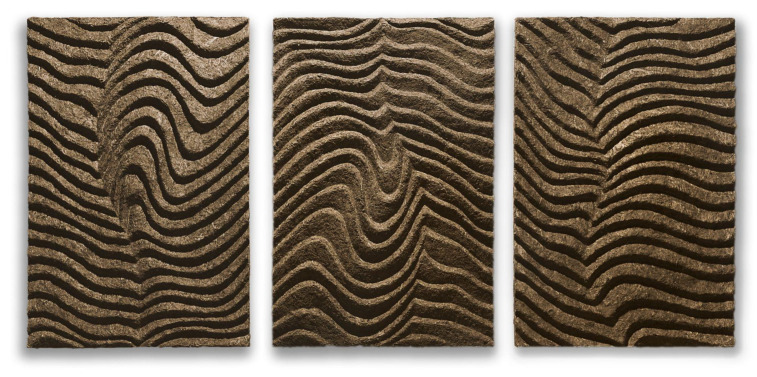
Sound absorption material from fillers (flax fiber and moss) with sapropel as a binder.

During studies, sound absorption materials were made by mixing dry fillers, flax fiber, and Sphagnum moss with sapropel, then putting the mass into a shape, compressing it, and drying it completely (Figure 3). These decorative acoustic wall panels were sound absorption, and decorative wall panels were made of ecological, breathable, and non-toxic composite materials. Panels could be connected and then hung onto the surface, holding on it only with its upper or upper and bottom parts. This prevented the wall or ceiling from being ruined with glue stains or drilling holes. These properties made the whole acoustic wall moveable, and they allowed the user to change the panel’s places and turn them upside down, thus changing the appearance of the acoustic wall.

### 3.2. Mechanical Strength Tests of Materials

**Figure 4:**
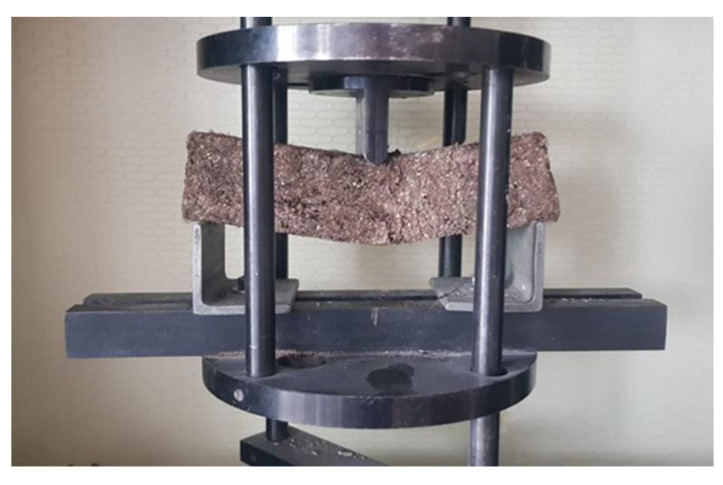
Test of bending resistance of a sample of sapropel, flax fiber, and vermiculite composite materials.

**Figure 5:**
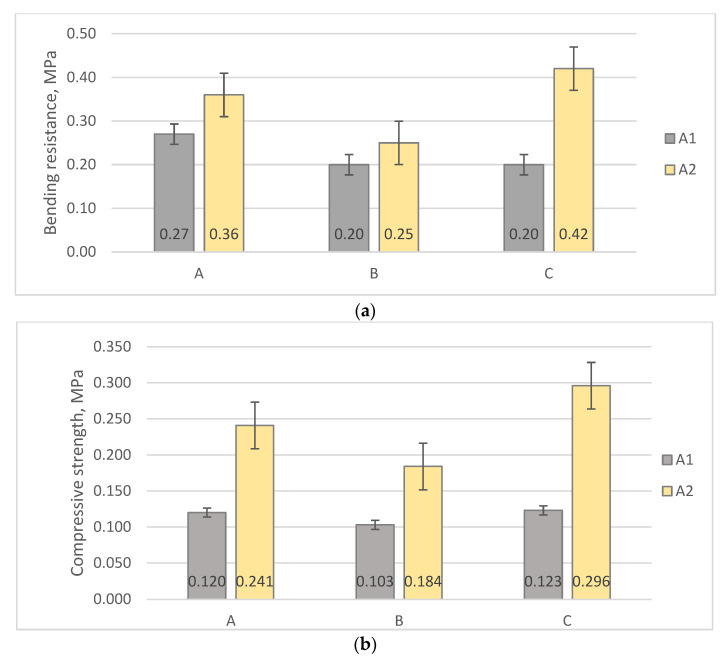
Mechanical properties of sapropel composite materials: (**a**) Bending resistance, MPa; (**b**) Compressive strength, MPa.

The bending resistance testing of composite materials is shown in Figure 4. The obtained bending resistance results showed that materials with vermiculite and flax fiber as fillers had higher bending strength than materials with moss and flax fiber as a filler (Figure 5a).

The highest result for bending resistance was C2 with 0.42 MPa, followed by A2 with 0.36 MPa. Compared to A2 and C2, the result of the bending strength of B2 was much lower, with 0.25 MPa. In addition, organic clay “ALINA LIFE ^TM^” was added to form B2. The lowest results for flexural strength were B1 and C1, which were 0.20 MPa.

By analyzing the obtained compressive strength results, it was concluded that the materials in which vermiculite and flax fiber were used as fillers had twice as high as the materials in which moss and flax fiber were used as fillers (Figure 5b).

By comparing the results of mechanical strength tests of composite materials developed in this study with the results obtained by testing composite materials using sapropel as a binder and wood fiber or birch wood sanding dust, or hemp shives and fibers as filler [8], it can be observed that sapropel, vermiculite, and flax fiber materials show higher bending and compressive strength than sapropel, hemp shives, and fiber materials, as well as sapropel and wood fiber materials. In turn, sapropel and wood birch sanding dust materials show higher compressive strength than obtained sapropel, vermiculite, and flax fiber materials–0.72 MPa. The study concluded that mixing wood fiber with a binder is more difficult than mixing slag with a binder, making it more difficult for the binder to enter the filler structure [8]. Similarly, in flax fibers, moss, and sapropel materials, when the binder is mixed with the filler, the binder penetrates more unevenly into the moss structure, thus creating air gaps that reduce mechanical strength. The location and interaction of the fibers, as well as the surface of the fibers, influence the mechanical properties of the materials [23].

Compressive strength is a very important property for materials that must be dimensionally stable for industrial purposes [23]. Acoustic materials, including sound-absorbing materials and structures, are commonly used in the construction of buildings as additional materials attached to the wall or ceiling surface to improve the environment (noise attenuation) and therefore do not require high mechanical strength [24]. In a study in which composite sound-absorbing composites were used using sawdust as a filler and polyurethane foam as a binder, the compressive strength tests ranged from 0.06 to 0.1 MPa, while the bending resistance tests ranged from 0.02 to 0.1 MPa [23]. 

By experimenting with binder-filler ratios in further studies, the mechanical properties of the resulting materials can be improved. Increasing the percentage of binder leads to a well-defined cell structure that gives greater mechanical strength compared to using a lower percentage of binder [23].

### 3.3. Sound Absorption Tests of Materials

Sound absorption tests were performed on materials marked A1, A2, B1, B2, C1, C2, D1, D2, E1, and E2, 25 mm thick and 10 mm thick, and two to three samples per parameter in the frequency ranging from 250 to 4000 Hz.

**Figure 6:**
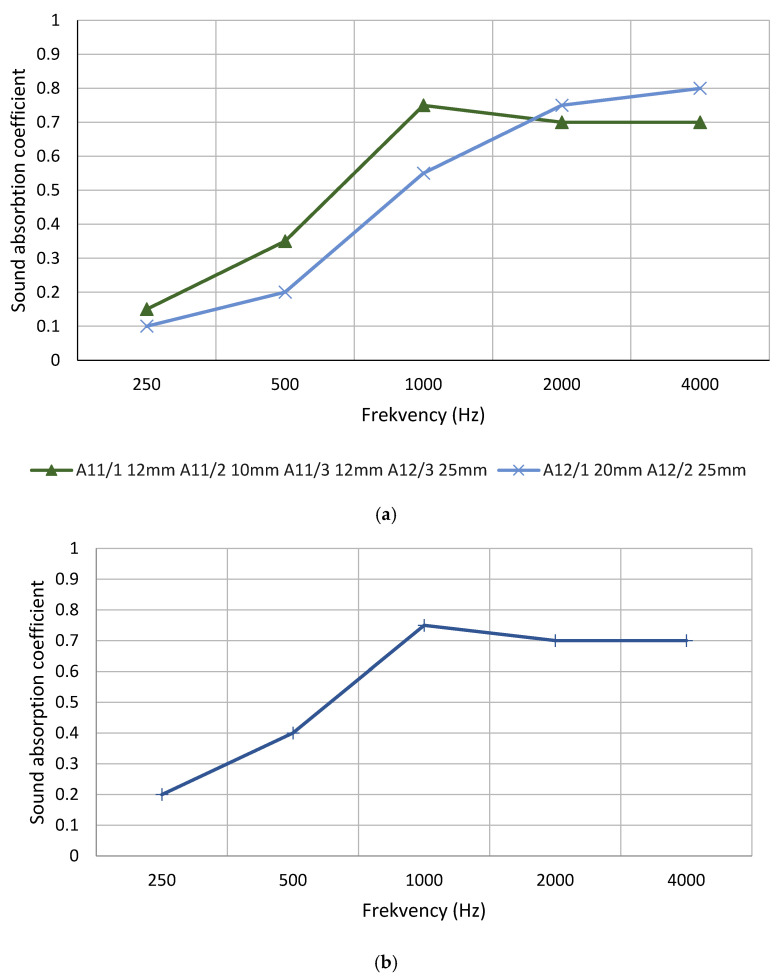
Sound absorption of composite materials without additives: (**a**) A1; (**b**) A2.

The resulting composites without additives had a sound absorption in lower frequencies and a high absorption in higher frequencies (Figure 6).

**Figure 7:**
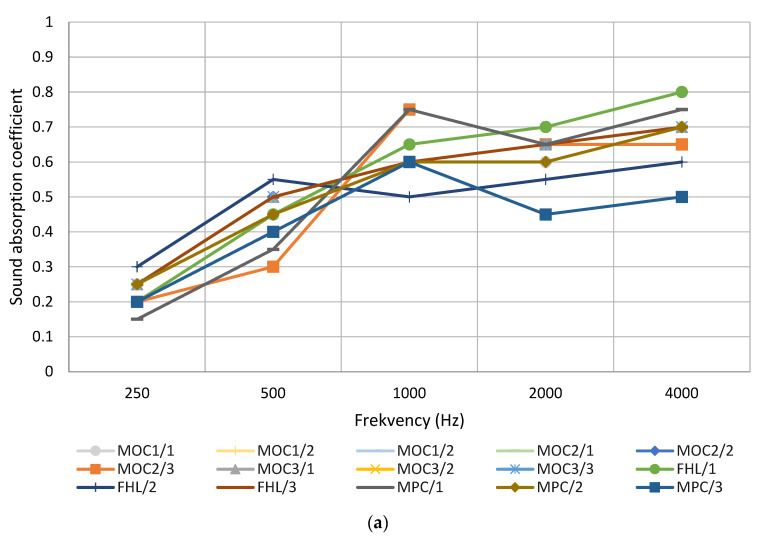

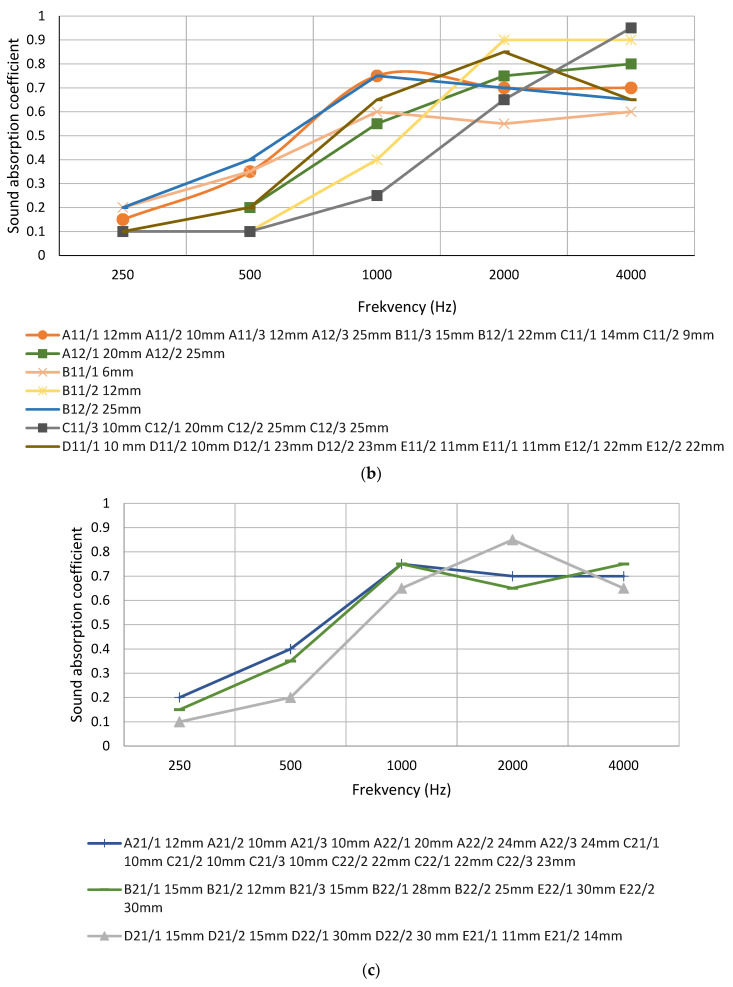
Sound absorption coefficient for composite materials: (**a**) Hemp composite materials; (**b**) Materials containing sapropel, moss, and flax; (**c**) Materials containing sapropel, vermiculite, and flax.

The created material samples A1 and A2, 22.7 mm and 23.3 mm thick, were compared with the previously studied [21] materials MOC1, MOC2, MOC3, FHL, and MPC. The thickest materials were compared because they corresponded more closely to the dimensions of the hemp composite materials. All hemp composite materials, except for MPC samples, were in the same sound absorption class (Figure 7). For samples containing hemp shives, the magnesium phosphate cement (MPC) sound absorption coefficient average was 0.6 in higher frequencies and corresponded to sound absorption class D, which is still absorbing. This could be due to porosity or brittleness, which made a bigger air gap with the test tube wall. Created materials A1 and A2 were in the same sound absorption class as hemp composite materials containing hemp shives, magnesium oxychloride (MOC) and hemp shives, magnesium phosphate cement (FHL)—absorbing (class D) in lower frequencies and highly absorbing (class C) in higher frequencies.

A comparison of the sound absorption coefficients of the two developed material types showed that materials in which vermiculite and flax fiber were used as fillers performed better and more stable than materials in which moss and flax fiber were used as fillers (Figure 6). 

One of the reasons for this difference could be the pore structure. In a study in which sound-absorbing composites were used using sawdust and polyurethane foam, the value of the coefficient in the sound-absorbing tests at 500 Hz increased with a 10% increase in polyurethane concentration. Polyurethane foam is commonly used as a sound-absorbing material due to its well-defined structure, which allows it to effectively absorb sound waves by means of air friction [23]. 

Recent studies have highlighted the importance of porosity in acoustic materials, more specifically, the distribution and structure of pore size, because when an acoustic wave reaches a porous surface, a significant portion of the acoustic energy is converted to heat, thus increasing noise reduction efficiency. In addition to the size of the porous cells and the size of the cell pore distribution, another important parameter for regulating the acoustic behavior is the interconnected porosity of the composites due to the cell structure of the binder and the fiber structure of the binder (longitudinal, regular fiber shape) [23].

The sound absorption capacity of a material is affected by factors such as the thickness of the material, the percentage of binder, the size and type of reinforcement material, and the air gap between the material and the rigid wall [23]. An important factor influencing the results of the sound absorption tests obtained is that the materials containing vermiculite fit more precisely to the walls of the test tube.

**Figure 8:**
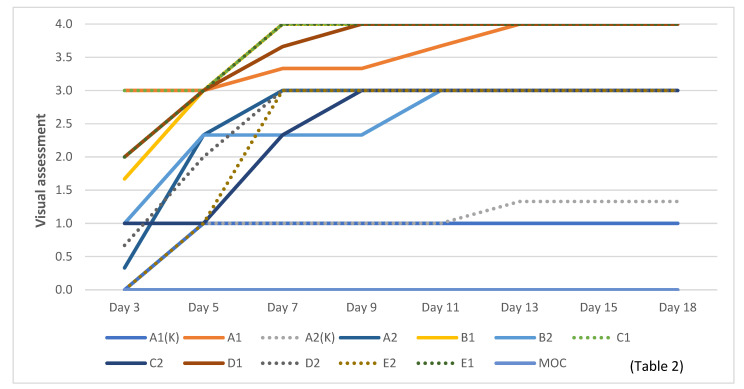
Rate of fungal spread in samples.

**Figure 9:**
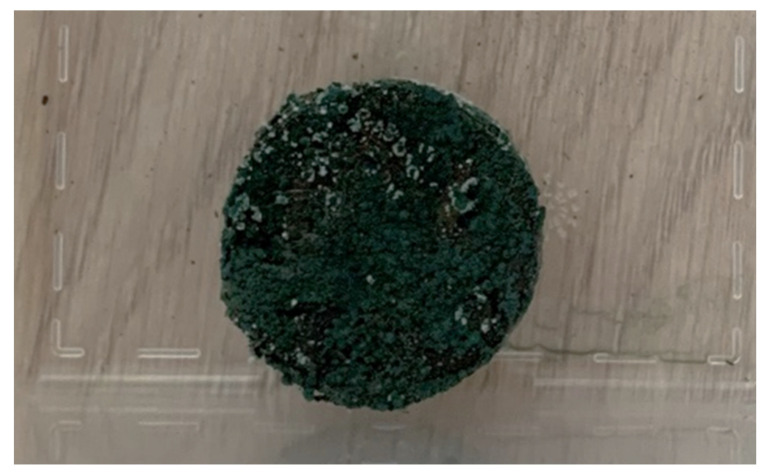
Fungi growth for sample D1 at the end of the incubation period.

By analyzing the sound absorption coefficient separately for composite materials containing moss, 25 mm thick samples often showed lower results than 10 mm thick materials (Figure 3, Figure 4, Figure 5, Figure 6, Figure 7, Figure 8 and Figure 9). The relationship between material thickness and frequency for mineral wool-based sound absorption plates has already been studied in the 1960s. It was concluded that as the thickness of the material decreases, the value of the maximum sound absorption coefficient moves to a higher frequency range [25]. It is confirmed that the thicker the sample, the greater the chance that gaps will form along the edges of the test tube. It can be concluded that the pore structure and gaps play a more important role in sound absorption than the thickness of the material. 

To improve the sound absorption of the materials, further studies can change the ratio of binder to filler or change the combination of fillers to improve the pore structure and make the edges of the material easier to handle, thus reducing the possibility of gaps between the material and the rigid wall.

By analyzing the sound absorption coefficient of materials containing vermiculite, it could be concluded that the difference in the thickness of the material does not significantly affect the sound absorption. Coated materials showed a slightly lower value, which could be explained by the fact that the coating reduces the porosity of the material, thus reducing the absorbency of the materials.

In a study conducted in Lithuania in 2016, where sapropel was used as a binder and hemp shives were used as a filler, materials with a compaction rate of 60%, 40%, and 20% were developed. Sound absorption tests were considered only for materials with a compaction level of 40%. Composites with a high level of compaction (60%) show almost no sound absorption properties. In these composites, the hemp fiber was well compacted and the surface was smooth. Therefore, sound waves could not get inside the composite and dissipate; instead, they were reflected from the composite surface. It is known that only open pores, which have a continuous air communication channel with the outer surface, provide good sound absorption properties [26].

Materials created during the study and hemp composite materials reached their sound absorbing peak at 1000 to 4000 Hz frequency. According to recent studies [27], raw hemp fiber has a sound absorption peak with a 0.95 sound absorption coefficient at 2000 Hz frequency. Other raw natural fibers, like pineapple leaf fiber, kenaf fiber, and coarse wool, reach their sound absorption peak at around 2000 Hz frequency, with a coefficient of 0.8 to 1. These fibers and composites developed in the study performed better than glass fiber, reaching absorption peak at 300 Hz, with a 0.9 sound absorption coefficient. 

### 3.4. Microbiological Resistance Tests of Materials

Microbiological stability tests were performed on sapropel, flax fibers, and vermiculite composites and sapropel, flax fibers, and moss composites without additional composition (Table 1), with ALINA 2% by weight or coating and biocide mass or coating by artificially inoculating *Aspergillus versicolor, Penicillium chrysogenum, Alternaria alternata, Cladosporium herbarum, Chaetomium* sp. and *Trichoderma asperellum*. The created materials without fungal inoculation K (control) were also kept in the climate chambers. The previously studied [21] composite materials MOC1, MOC2, and MOC3 were also incubated for comparison (Table 2).

#### 3.4.1. Observations of the Rate of Mold Spread

Mold growth depends on various environmental factors. The main factor is the availability of water. To describe the state of water in the environment, relative humidity is usually used, which is the ratio between the actual water vapor content per m3 of air and the maximum amount of water vapor (saturated content) that can be present in each volume at a given temperature [28]. Under stable conditions, fungal growth begins at about 80% of relative humidity [29]. The minimum acceptable relative humidity is 77% and the optimal is 97% [30]. In turn, the optimum temperature for many fungal species is between 20 and 30 °C [31]. The substrate (or material) is also important. Distribution depends on the number of nutrients available and the porosity and roughness of the material [32]. 

By analyzing the distribution schedule of fungi, it was observed that molds spread most rapidly to A1, B1, C1, D1, and E1 materials (Figure 8). All of these were materials that used moss and flax fiber as fillers. 

Materials with vermiculite and flax fiber as fillers had a slower spread of fungi than those containing moss. Materials that used moss as a filler were more porous than those that used vermiculite. In a study comparing the microbiological resistance of birch wood slate, sapropel composites with microbiologically stable sphagnum moss, and peat sapropel composites, it was found that the denser the material, the more difficult it is to multiply. In more porous materials fungi begin to grow faster. All fungi used in the experiment were visible on cellulose residues [8]. It could be concluded that the substrate and nutrients played an important role in the spread of the fungi in the experiment.

For MOC materials, the fungal spread was not observed visually. The pH of the hemp clumps, magnesium oxychloride materials, was much higher (9.76) [21] than the developed sapropel-filler materials, as seen in Figure 8.

Materials A1 and C1, already in the first visual evaluation, were evaluated with three, which was assigned if macroscopic growth of fungi was detected (Table 4). At the next evaluation, two days later, such an increase was also observed for B1, D1, and E1 materials (Figure 8).

At the end of the incubation period, materials A1, B1, C1, D1, and D2 were the only ones to have achieved a visual assessment four (Figure 8), which was granted if the macroscopic growth covered > 80% of the material surface (Table 4), as seen on the sample. D1 in Figure 9.

#### 3.4.2. Microscopic Identification of Dominant Mold Colonies

At the end of the 16-day incubation period, the samples were visually evaluated for the last time and then examined microscopically. The latest results of the visual assessment and the genera of microscopically identified molds are marked in Table 6. 

Fungal colonies were detected microscopically in all incubated samples except MOC1 (K), MOC2 (K), and MOC3 (K).

*Trichoderma* molds were most frequently found in samples from artificially inoculated fungi (Table 6). These fungi could not be distinguished microscopically due to a large number of spores, but fungi of this genus could be distinguished macroscopically on the samples, which could be observed visually, for example, in sample D1 (Figure 9). Spores of molds of the genus *Trichoderma* were characterized by a dark green color (Figure 2).

**Figure 10:**
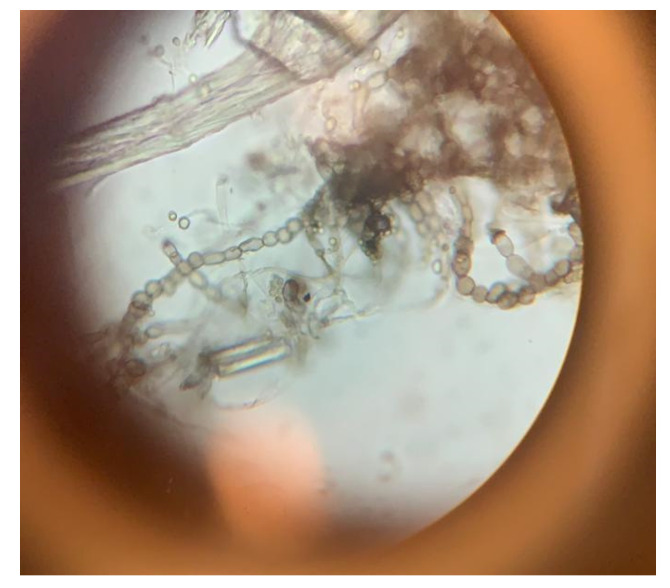
Aureobasidium mold sample A1 in a microscope.

Molds of the genus *Aureobasidium* were often found in the samples. *Cladosporium* and *Mucor* species were found in the samples of A1 (Figure 10) (Table 6). Fungi of the genus Mucor were not artificially inoculated on the samples (Figure 2), so they were already present in the samples and had started to multiply on their own.

Based on the microbiological resistance tests, it could be concluded that the studied materials had a high risk of biodegradation because the rapid growth of mold colonies was the first indication that the materials had begun to degrade and lose their properties. The next organism after fungi that can start to degrade materials is bacteria, but they need more moisture [8]. 

The most rapid degradation was observed in sapropel, flax fiber, and moss materials, which were completely enveloped by fungi two days after inoculation (Figure 8). Although sapropel, flax fiber, and vermiculite materials did not spread as rapidly as moss-containing materials, they were also relatively rapidly absorbed by fungi compared to hemp shives and magnesium oxychloride, for which the fungi were only detected microscopically.

## 4. Conclusions

Using natural fillers (vermiculite, flax fiber, and sphagnum moss) and sapropel as a binder can create environmentally friendly composite materials, adapting them to the needs of the application. The created composite materials are visually characterized by unique natural design properties, and the mechanical strength indicators are in accordance with the sound-absorbing materials used in construction as additional elements for noise reduction.

Composite materials showed good sound absorption test results according to normal absorption and are suitable for high-frequency noise attenuation. The sapropel, flax fiber, and vermiculite materials and sapropel, flax fiber, and sphagnum moss materials and hemp materials are suitable materials for sound-absorbing panels and other ancillary elements of buildings. In addition, after improving their mechanical strength, they are also basic sound-absorbing elements. By improving the sound absorption materials of sapropel and adjusting their composition and composition ratio, it is possible to further improve the sound absorption performance and create unique, environmentally friendly, high-quality sound absorption materials for use in urban noise suppression.

The formed sound-absorbing composite materials have an increased content of organic matter and a pH corresponding to the growth of fungi (5.7–7.0); therefore, they are vulnerable to microbiological degradation and, thus, must be treated with antimicrobial additives.

## Figures and Tables

**Table 1:** Characteristics of sapropel samples.

Origin	Color	Structure	pH	Org., %	Moisture, %
Lake Pilvelis	Dark brown with a greenish tinge	Shaggy	6.76	84.51	94.99
Lake Piksteres	Brownish black	Viscose	6.84	83.00	97.00

**Table 2:** Composition, basic composition, and additives of composite materials created in the experiment.

Basic Composition	
Sapropel, moss, flax	1
Sapropel, vermiculite, flax	2
**Additives**	
No additives	A
ALINA 2% in mass	B
BIOCIDE 1% in mass	C
BIOCIDE 1% coating	D
ALINA 2% coating	E
Control (without inoculation of fungi)	(K)

**Table 3:** Composition of previously studied environmentally friendly composite materials and their designations.

The Composition	
Hemp shives, magnesium oxychloride cement 20%	MOC1
Hemp shives, magnesium oxychloride cement 50%	MOC2
Hemp shives, magnesium oxychloride cement 100%	MOC3
Hemp shives, formulated hydraulic lime	FHL
Hemp shives, magnesium phosphate cement	MPC

**Table 4:** Fungal colony growth expert rating scale.

Coefficient	Visual Assessment of Fungal Colony Growth
0	No fungal growth was observed microscopically
1	Fungal growth has been observed microscopically
2	Fungal growth observed microscopically covering the entire surface of the sample
3	Macroscopic growth of fungi (visible to the naked eye) ≤80% of the material surface
4	Macroscopic growth covering >80% of the material surface

**Table 5:** Characteristics of composite materials.

Sample	Material Description	Raw Materials	Ratio *	Density, kg/m^3^	pH
A1	Lightweight, keeps shape well. Easy to break. Difficult to break into smaller fractions. Difficult to grind, especially thinner specimens. Moss in the material is very visible. Dark brown.	Flax fiber, sphagnum moss, sapropel 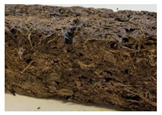	1:6	334	5.98
B1	Same as A1.	Flax fiber, sphagnum moss, sapropel + ALINA 2% by weight	1:6	334	6.22
C1	Same as A1.	Flax fiber, sphagnum moss, sapropel + Biocide 1% by weight	1:6	306	5.70
D1	As lightweight as A1, but the coating provides better shape stability, and no moss is visible in the material.	Flax fiber, sphagnum moss, sapropel + Biocide 1%, sapropel coating	1:6		5.74
E1	Same as D1.	Flax fiber, sphagnum moss, sapropel + ALINA 2%, sapropel coating	1:6		6.45
A2	Lightweight. Keeps shape well. Hard to break. Easy to break into smaller fractions. Easy to sand. Dark brown. Vermiculite gives shine.	Flax fiber, vermiculite, sapropel 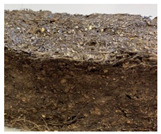	1:6	344	6.73
B2	Same as A2.	Flax fiber, vermiculite, sapropel + ALINA 2% by weight	1:6	389	6.95
C2	Same as A2.	Flax fiber, vermiculite, sapropel + Biocide 1% by weight	1:6	357	6.51
D2	As lightweight as A2. Harder to break than A2. The coating hides the vermiculite.	Flax fiber, vermiculite, sapropel + Biocide 1%, sapropel coating	1:6		6.46
E2	Same as D2.	Flax fiber, vermiculite, sapropel + ALINA 2%, sapropel coating	1:6		6.49

* filler to binder mass ratio

**Table 6:** Microscopically detected mold genera in samples.

Sample	Visual Assessment	Mold Colonies (Genus)
A1 (K)	1	*Trichoderma*
A1	4	*Aureobasidium; Penicillium*; *Mucor*; *Chaetomium*; *Trichoderma* (dominant)
A2 (K)	1	*Trichoderma*
A2	3	*Aureobasidium; Penicillium*; *Mucor*; *Trichoderma* (dominant)
B1	4	*Trichoderma*
B2	3	*Trichoderma*
C1	4	*Trichoderma*
C2	3	*Trichoderma; Cladosporium*; *Aureobasidium*
D1	4	*Trichoderma*
D2	3	*Trichoderma*; *Cladosporium*
E1	4	*Trichoderma*
E2	3	*Trichoderma*
MOC1 (K)	0	No fungi spread was observed
MOC2 (K)	0	No fungi spread was observed
MOC3 (K)	0	No fungi spread was observed
MOC1	0	*Aspergillus* (dominant); *Penicillium*
MOC2	0	*Cladosporium* *Aspergillus*
MOC3	0	*Aspergillus*

## Data Availability

Not applicable.
